# Sustainability of a Motor Control Exercise Intervention: Analysis of Long-Term Effects in a Low Back Pain Study

**DOI:** 10.3389/fspor.2021.659982

**Published:** 2021-07-20

**Authors:** Anne-Katrin Puschmann, Chiao-I Lin, Pia-Maria Wippert

**Affiliations:** ^1^Sociology of Medicine and Psychobiology, Department of Physical Activity and Health, University of Potsdam, Potsdam, Germany; ^2^Faculty of Health Sciences Brandenburg [University of Potsdam, the Brandenburg Medical School Theodor Fontane and the Brandenburg University of Technology Cottbus – Senftenberg], Senftenberg, Germany

**Keywords:** MiSpEx, low back pain, long-term effects, multidisciplinary intervention, sustainability

## Abstract

Development of chronic pain after a low back pain episode is associated with increased pain sensitivity, altered pain processing mechanisms and the influence of psychosocial factors. Although there is some evidence that multimodal therapy (such as behavioral or motor control therapy) may be an important therapeutic strategy, its long-term effect on pain reduction and psychosocial load is still unclear. Prospective longitudinal designs providing information about the extent of such possible long-term effects are missing. This study aims to investigate the long-term effects of a homebased uni- and multidisciplinary motor control exercise program on low back pain intensity, disability and psychosocial variables. 14 months after completion of a multicenter study comparing uni- and multidisciplinary exercise interventions, a sample of one study center (*n* = 154) was assessed once more. Participants filled in questionnaires regarding their low back pain symptoms (characteristic pain intensity and related disability), stress and vital exhaustion (short version of the Maastricht Vital Exhaustion Questionnaire), anxiety and depression experiences (the Hospital and Anxiety Depression Scale), and pain-related cognitions (the Fear Avoidance Beliefs Questionnaire). Repeated measures mixed ANCOVAs were calculated to determine the long-term effects of the interventions on characteristic pain intensity and disability as well as on the psychosocial variables. Fifty four percent of the sub-sample responded to the questionnaires (*n* = 84). Longitudinal analyses revealed a significant long-term effect of the exercise intervention on pain disability. The multidisciplinary group missed statistical significance yet showed a medium sized long-term effect. The groups did not differ in their changes of the psychosocial variables of interest. There was evidence of long-term effects of the interventions on pain-related disability, but there was no effect on the other variables of interest. This may be partially explained by participant's low comorbidities at baseline. Results are important regarding costless homebased alternatives for back pain patients and prevention tasks. Furthermore, this study closes the gap of missing long-term effect analysis in this field.

## Introduction

Becoming chronic pain with annual costs of 49 billion euros and 90,000 official diagnoses (Wenig et al., 2009), back pain is the third most frequent acute diagnosis in the Federal Republic of Germany (Statistisches-Bundesamt, 2015). Along with painkillers, physiotherapeutic measures are the most frequently prescribed therapy form (Renker et al., 2009). Clinically relevant back pain is particularly pronounced in people with inadequately trained back muscles on the one hand and in people with high levels of stress such as in top-class sports on the other.

Deficits on the neuronal, muscular and/or structural level are usually cited as decisive for the development of complaints (Yahia et al., 2011; Willigenburg et al., 2013). Studies revealed that chronic back pain patients also show an increased sensitivity to pain and altered pain processing mechanisms, whose causes can primarily be attributed to central nervous mechanisms from a biopsychological point of view (Brooks et al., 2002; Wippert and Wiebking, 2018). The roots of chronification can be traced back to cortical reorganizations in the patient's brain, in particular to the individual development of pain memory and a disturbance in the deletion of pain-related memories (Zhuo, 2018).

In addition to classical conditioning mechanisms, affective and social factors have an effect on memory processes and neuroplasticity, so that depression, anxiety and low self-efficacy can contribute to maintaining pain through their limiting effect on compliance (Nicholas et al., 2011; Wippert and Wiebking, 2018). The integration of behavioral therapy approaches into classical physiotherapy and movement therapy programs can potentially break off back pain related associations as a prerequisite for chronification processes and enable long-term therapeutic success (Linton and Shaw, 2011; Wippert et al., 2015).

As postulated by a recent review from Wilson (Wilson, 2017), more research about the long-term effects of multidisciplinary programs is highly necessary, since there is little evidence about the sustaining effects of such programs on low back pain (Flor, 2011; Saragiotto et al., 2016). In order to fill this gap, the here reported results provide a follow-up to an intervention study with uni- and multidisciplinary interventions to record long-term effects. The multidisciplinary program aimed at reinforcing and classical conditioning processes and included elements of stress reduction and cognitive behavioral therapy. The novelty of the unimodal therapy was a short-time motor control training program that could be performed at home with only little additional material. The here presented paper addresses the following key questions:

1. Do long-term effects on pain intensity and disability differ between uni- vs. multidisciplinary motor control exercise interventions?
2. Is the multidisciplinary exercise intervention more sustainable than uni-disciplinary exercises in its effects on (a) pain related cognitions? (b) stress load?

## Materials and Methods

### Study Design

The initial motor control exercise study was a multicenter 3-armed randomized controlled trial including a 12-week intervention. Participants were allocated into unimodal or multidisciplinary intervention (*nblock* = 18, basis 1:1, www.randomization.com). The unimodal intervention was a motor control training (SMT, three times/week, 30 min, four exercises and 12 activity levels). The multidisciplinary intervention SMT + BT was SMT combined with a behavioral therapy module (BT, includes body scan relaxation, partner-education film and cognitive distraction tasks additional to SMT) (Wippert et al., 2015, 2019, 2020). The study was registered as a clinical trial 05/16/2013 in the German Clinical Trial Register with the identification number: DRKS00004977 (https://www.drks.de/drks_web/navigate.do?navigationId=trial.HTML&TRIAL_ID=DRKS00004977) (Wippert et al., 2015, 2019, 2020).

**Figure 1:**
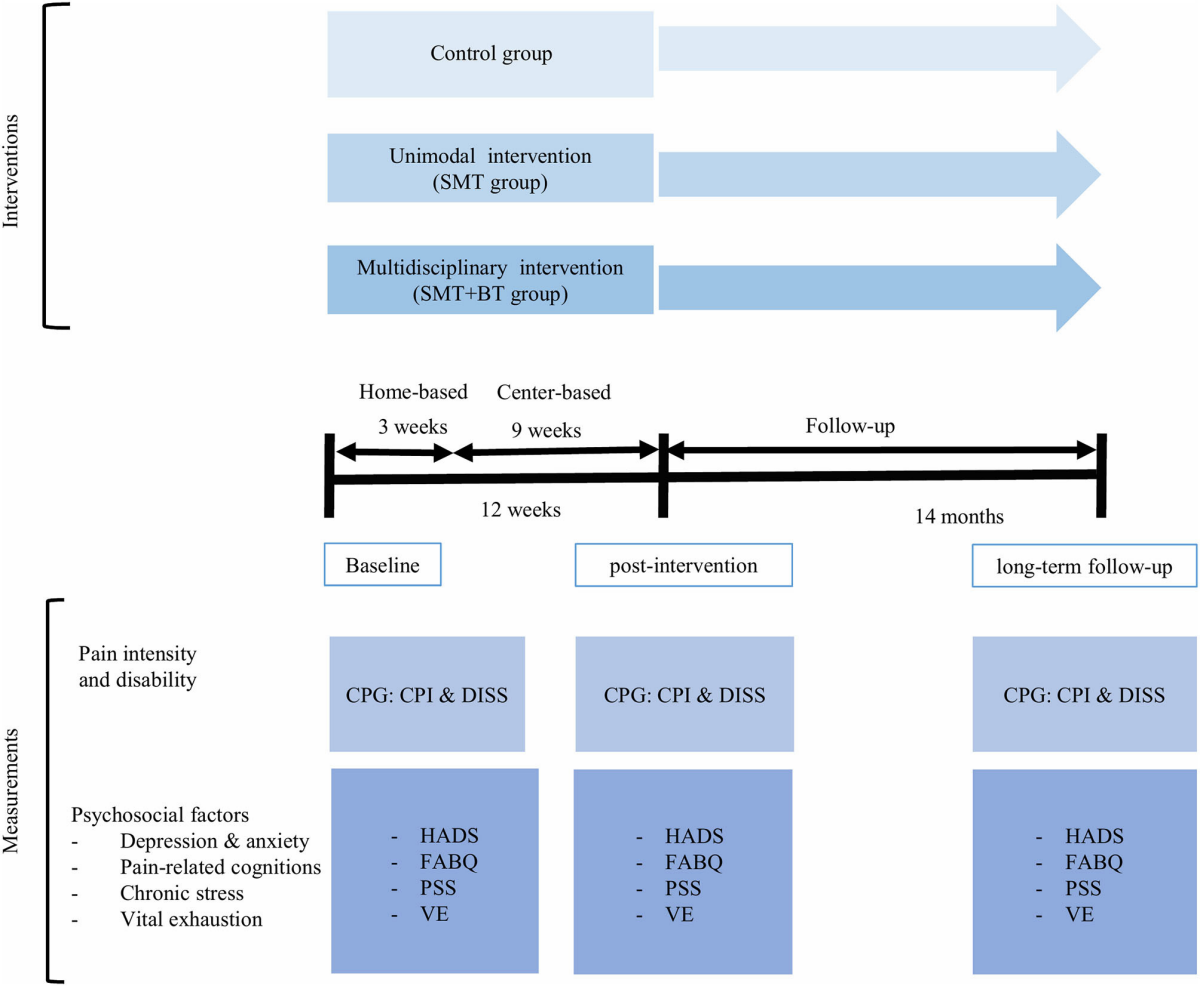
Study design: SMT: sensorimotor training; SMT + BT: sensorimotor training and behavioral therapy module; CPG: Chronic Pain Grade questionnaire; CPI: Characteristic Pain Intensity; DISS: Disability; HADS: Hospital Anxiety and Depression Scale; FABQ: Fear Avoidance Beliefs Questionnaire; PSS: Perceived Stress Scale; VE: Maastricht Vital Exhaustion Questionnaire 96 Short Form (VE).

The intervention was conducted center-based over a course of three weeks and was subsequently continued on a home-based level for an additional nine weeks. In order to control the exercise execution, at the center-based training, a sports- and physiotherapists supervised participants' training and at the home-based training, participants performed the exercise with an audio-guided DVD and wrote exercise diaries (Wippert et al., 2015). After the first baseline measurements, intervention effects were measured after 12 weeks of intervention (post-intervention). The long-term effects presented here were assessed 14 months after the end of the intervention (long-term follow-up) (Figure 1).

The main objective criteria for recording long-term effects of the unimodal and multidisciplinary intervention were pain intensity and pain-related disability as well as psychosocial factors such as psychophysiological stress, anxiety, depression, and pain-related cognitions. All participants agreed to answer the survey in advance and thereby declared their consent to participate in the study. All surveys were designed and implemented in accordance with the principles of the Declaration of Helsinki and approved by the independent Ethics Committee of the University of Potsdam, Germany (Ethics Vote of the University of Potsdam, No. 19/2015).

### Study Sample

In total *n* = 154 participants were recruited from the study center at the University of Potsdam, and completed were contacted by telephone six months after completion of the last measurements, and asked to take part in this additional follow-up survey. Table 1 presents participants' characteristics at the baseline. Participants initiated the intervention at different time points. The research center contacted participants six months after all measurements were completed. Therefore, the participants who started intervention earlier have a longer follow-up period, and the participants who started later have a shorter follow-up period. On average, the long-term follow-up period was 14 months. The study sample is a subsample from the main intervention study (MiSpEx DRKS00004977). The sample size calculation of the main study was based on an unpublished dataset (*a* ≤ 0.05; *1-*β = 0.999, drop out 30%, power analysis by G^*^Power, 36 effect size *f* = 0.25, sample size: *n* = 600).

**Table 1:** Participants' characteristics at the baseline.

	**Control group**		**SMT**		**SMT + BT**		
	***M***	***SD***	***M***	***SD***	***M***	***SD***	**Differences among groups**
Age (year)	35	12	36	13	34	12	*p* > 0.05
Height (cm)	172	8	173	8	174	10	*p* > 0.05
Weight (kg)	69	13	72	15	73	14	*p* > 0.05
Duration of physical activity per month (min)	900	805	748	990	876	939	*p* > 0.05
Sex (Men/Women)	14/31		20/30		22/25		*p* > 0.05
Medication taken	41%		43%		34%		*p* > 0.05

*M: mean; SD: standard deviation; SMT: sensorimotor training; SMT+ BT sensorimotor training and behavioral therapy module*.

Inclusion criteria were age between 18 and 65 years with intermittent back pain (defined as a minimum score of two on a numerical rating scale (0—no pain, 10—worst pain). Exclusion criteria were 1) suffering from acute infections (past seeven days); 2) current pregnancy; 3) not being able to stand upright independently, 4) not being able to move independently from a lying position to a standing position; 5) being unable to complete a questionnaire on their own (without outside help); 6) a diagnosis that excludes physical activity; and 7) acute back pain that has occurred within the last seven days. All participants received written and oral information and having signed an informed consent form, before participating.

### Instruments

Long-term effects were measured by a questionnaire-based follow-up survey. *Characteristic pain intensity* (CPI) and *pain related disability* (DISS) of the previous three months were assessed with the Chronic Pain Grade questionnaire (CPG) (Korff et al., 1992).

The presence of *Anxiety* and *Depression* were determined using the Hospital and Anxiety Depression Scale (HADS) (Zigmond and Snaith, 1983). *Pain-related cognitions* were measured with the Fear Avoidance Beliefs Questionnaire (FABQ: subscales work and activity) (Pfingsten et al., 1997). *Chronic stress* was recorded using the German version of the Perceived Stress Scale (PSS) (Cohen et al., 1983). The German short version of the Maastricht Vital Exhaustion Questionnaire (VE) (Appels et al., 1987) was used to quantify vital exhaustion.

### Statistical Analysis

Three measurement points were included in the statistical analysis (baseline, post-intervention, long-term [14 months] follow-up). Statistical analysis was performed using IBM SPSS Statistics 24.0. Repeated measures mixed ANCOVA (*time*: baseline, post-intervention, long-term follow-up; *group*: control group, unimodal, multidisciplinary) were calculated to determine the long-term effects of the intervention on characteristic pain intensity and pain related disability as well as on the psychosocial factors (general anxiety and depression; pain related cognitions: FABQ scales work and activity; PSS and VE). All calculations were adjusted for age, sex, and time difference post-intervention to follow-up. Significance was set at a level of α = 0.05. Since groups were compared with regard to changes within the variables of interest, time^*^group interaction effects were reported, including η_*p*_^2^ as a measure of effect size (Bühner and Ziegler, 2017). If significance was detected, further analyses were conducted group wise to determine where exactly the change took place. Missing data were dealt with based on questionnaire manuals and American Psychology Association guidelines. Missing cases (e.g. missing questionnaire at measurement point) were not analyzed (APS, 2020).

## Results

### Descriptive

In total, *n* = 84 subjects participated in the follow-up survey (response-rate = 54.5%). 59.5% of the subjects were female. Average age of the sample population was 34.8 years (*SD* = 11.7). The groups were nearly equally distributed with *n* = 29 subjects being part of the control group, n = 28 of the SMT group and n = 27 of the SMT+BT group. Table 2 showed the characteristics of participants who attended follow-up and who did not at the baseline, post-intervention, and long-term follow-up (14-month).

**Table 2:** The characteristics of participants who attended follow-up and who dropped out at the baseline and after intervention.

	**Participants attending follow-up (*****n*** **= 84)**				**Participants did not attend follow-up (*****n*** **= 58)**				
	**Baseline**		**After intervention**		**Baseline**		**After intervention**		**Differences between groups**
	***M***	***SD***	***M***	***SD***	***M***	***SD***	***M***	***SD***	
Age (year)	35	12	35	12	34	12	34	12	*p* > 0.05
Height (cm)	173	8	173	9	173	10	171	10	*p* > 0.05
Weight (kg)	71	13	71	14	72	15	72	15	*p* > 0.05
Duration of physical activity per month (min)	893	930	910	990	775	883	819	936	*p* > 0.05
Sex (Men/Women)	34/50		26/43		22/36		13/29		*p* > 0.05
Medication taken	42%		40%		43%		42%		*p* > 0.05
Analgesic medication taken	21%						11%		*p* > 0.05

*M: mean; SD: standard deviation*.

Baseline and follow-up values of pain intensity, disability and psychosocial variables are displayed in Table 3. At the long-term follow-up, of all groups, subjects of the SMT group reported the lowest CPI (M[SD] = 25.8 [14.8] in comparison to the SMT+BT group (M[SD] = 26.4 [23.5]) and controls (M[SD] = 28.3 [18.9]. The lowest disability score at follow-up was reported by the SMT+BT group (M[SD] = 8.1 [14.5] compared to the SMT group (M[SD] = 9.1 [11.2] and controls (M[SD] = 13.7 [17.4]).

**Table 3:** Descriptive values *(M, SD)* of the study sample at baseline and long-term follow-up.

	**Control group**						**SMT**						**SMT + BT**					
	**baseline**		**post-intervention**		**long-term follow-up**		**baseline**		**post-intervention**		**long-term follow-up**		**baseline**		**post-intervention**		**long-term follow-up**	
**Construct to be measured (Range)**	***M***	***SD***	***M***	***SD***	***M***	***SD***	***M***	***SD***	***M***	***SD***	***M***	***SD***	***M***	***SD***	***M***	***SD***	***M***	***SD***
Characteristic pain intensity(0–100)^†^	26.8	16.8	21.4	15.3	28.3	18.9	33.3	17.4	23.5	16.4	25.8	14.8	27.0	22.3	21.5	21.3	26.4	23.5
Disability score (0–100)^†^	9.6	15.6	4.9	8.8	13.7	17.4	17.9	18.1	12.9	17.3	9.1	11.2	15.8	20.7	14.9	20.7	8.1	14.5
Depression (0–21)^‡^	3.1	2.4	3.4	3.0	3.3	2.8	3.8	3.2	2.9	2.6	2.5	2.1	3.5	2.8	3.2	3.3	3.2	3.2
Anxiety (0–21)^‡^	5.4	2.3	6.0	3.5	5.7	3.3	5.1	3.8	5.2	4.0	5.4	3.5	4.6	2.4	4.4	2.6	4.5	2.8
Work: belief that back pain is related to work (0–30^§^	12.0	6.2	10.0	7.0	11.3	7.2	12.6	6.3	11.4	7.1	11.5	6.6	11.2	5.4	13.8	7.2	10.6	7.0
Activity: belief that back pain is related to physical activity (0–30)^§^	9.3	7.7	7.3	6.7	7.6	7.4	9.5	7.0	8.0	6.6	6.8	7.1	6.8	6.8	7.6	7.6	6.4	8.1
Perceived stress (0–40)^¶^	16.7	5.9	16.5	5.9	15.8	6.8	16.6	5.5	15.0	6.1	12.4	5.5	14.9	6.1	15.5	6.0	12.3	6.1
Vital exhaustion (0–18)^#^	8.6	5.0	7.9	5.0	7.4	4.5	7.5	4.9	5.8	4.7	6.4	4.9	6.4	5.3	5.6	5.0	5.9	4.7

† *Chronic Pain Grade questionnaire (CPI: Characteristic Pain Intensity, DISS: Disability)*;

‡ *Hospital Anxiety and Depression Scale (HADS-D)*;

§ *Fear Avoidance Beliefs Questionnaire (FABQ-D)*;

¶ *Perceived Stress Scale (PSS)*;

# *Maastricht Vital Exhaustion Questionnaire – Short Form (VE); SMT: sensorimotor training, SMT + BT: sensorimotor training and behavioral therapy module; M: mean, SD: standard deviation*.

The lowest depression score at follow-up was reported by the SMT group (M[SD] = 2.5 [2.1], compared to the SMT+BT group (M[SD] = 3.2 [3.2]) and controls (M[SD] = 3.3 [2.8]). The lowest anxiety score was reported by the SMT+BT group (M[SD] = 4.5[2.8]), compared to the SMT group (M[SD] = 5.4 [3.5]) and controls (5.7[3.3]).

### Long-Term Effects on Pain Intensity and Disability

The repeated measures ANCOVA revealed a significant time^*^group effect when comparing groups with respect to DISS at baseline, post intervention and long-term follow-up ($F_{time^{*}group}$_(4,118)_ = 3.931; *p* = 0.005*;* η_*p*_^*2*^ = 0.118). Significant long-term changes are present in the unimodal group between post-intervention and long-term follow-up (*F*_*time*__(2,38)_ = 4.591; *p* = 0.016; η_*p*_^*2*^ = 0.195; pairwise comparisons, Bonferroni corrected: Δ_*post*−*interv, baseline*_ = −7.17 [95%CI: −15.61; 1.28]; Δ_*follow*−*up, post*−*interv*_ = −7.05 [95%CI: −13.20; – 0.90]). The change in the multidisciplinary group did not reach statistical significance (*F*_*time*__(2,32)_ = 1.441; *p* = 0.252; η_*p*_^*2*^ = 0.083; pairwise comparisons, Bonferroni corrected: Δ_*post*−*interv, baseline*_ = 2.52 [95%CI: −8.36; 13.40]; Δ_*follow*−*up, post*−*interv*_ = −7.64 [95%CI: −20.94; 5.67]) nor did the change in the control group (*F*_*time*__(2,40)_ = 0.421; *p* = 0.660; η_*p*_^*2*^ = 0.021; pairwise comparisons, Bonferroni corrected: Δ_*post*−*interv, baseline*_ = −4.82 [95%CI: −11.32; 1.69]; Δ_*follow*−*up, post*−*interv*_ = 6.25 [95%CI: −2.23; 14.72]).

The time^*^group effect for CPI did not reach statistical significance ($F_{time^{*}group}$_(4,120)_ = 0.997; *p* = 0.412; η_*p*_^*2*^ = 0.032).

### Long-Term Effects on Psychosocial Variables

Regarding anxiety and depression, no significant time^*^group effect could be found (depression: $F_{time^{*}group}$_(4,110)_ = 2.267, *p* = 0.067, η_*p*_^*2*^ = 0.076; anxiety: Mauchly's *W* = 0.856, *p* = 0.015, Greenhouse-Geisser: $F_{time^{*}group}$_(3.5, 97.9)_ = 0.283, *p* = 0.866, η_*p*_^*2*^ = 0.010).

There was no significant time^*^group effect in perceived stress (Mauchly's *W* = 0.880, *p* = 0.032; Greenhouse-Geisser: $F_{time^{*}group}$_(3.6, 98.2)_ = 1.130, *p* = 0.345, η_*part*_^*2*^ = 0.039) and vital exhaustion ($F_{time^{*}group}$_(4,118)_ = 0.561, *p* = 0.692, η_*p*_^*2*^ = 0.019). There was also no significant time^*^group effect in pain related cognitions (FABQ work: $F_{time^{*}group}$_(4,116)_ = 1.837, *p* = 0.126, η_*part*_^*2*^ = 0.060; FABQ activity: $F_{time^{*}group}$_(4,114)_ = 1.874, *p* = 0.120, η_*p*_^*2*^ = 0.062).

## Discussion

The present study is—to our knowledge—one of the first studies analyzing long-term effects of uni- and multimodal exercise programs on low back pain. It is assumed that the most effects will only be of short-term duration (Choi et al., 2010), but indeed, there is a lack of knowledge about the extent of effect changes over time after an exercise intervention. The main purpose of the current study was therefore to investigate the difference of long-term effects from uni- and multidisciplinary motor control exercise interventions on pain intensity, disability and psychosocial variables.

In view of the primary research question, the data indicates a superior long-term effect of motor control exercise training on pain related disability. Subjects of the SMT group could benefit from a long-term effect of the motor control training on pain-related disability, with a large effect size of η_*p*_^*2*^ = 0.195 (corresponding *Cohen's f* = 0.49), whereas the change of disability in the multidisciplinary group missed statistical significance, although pointing to a medium sized effect (η_*p*_^*2*^ = 0.083, corresponding *Cohen's f* = 0.301). Despite these effects on disability, the effect of the two interventions showed no group difference of long-term effect on pain intensity. These results are consistent with Wilson's findings that the benefits of multidisciplinary approaches for pain patients seem to be clearest for physical and psychological functioning, whereas the effects on pain intensity seem to be rather low and short termed (Wilson, 2017). Both treatments seem to promote physical functioning, even under the influence of chronic pain.

Considering the second research question about the differences between the interventions on cognitions, the results do not support a long-term effect of the multidisciplinary approach on anxiety and depression as well as pain-related cognitions. It should be noted that the change in depression—although missing statistical significance—showed a medium effect size (η_*p*_^*2*^ = 0.076; corresponding *Cohen's f* = 0.287). This direction is supported by recent findings on the positive influence of exercise interventions on depression, regardless of the kind of exercise intervention (Schuch et al., 2015; Roy et al., 2018; Irandoust et al., 2019). Subjects of the SMT+BT group reported their pain least at work and physical activity, this result is also missing statistical significance, although displaying a medium sized effect (η_*p*_^*2*^ = 0.06, corresponding *Cohen's f* = 0.25). There were no statistically significant group differences in perceived stress and vital exhaustion.

Since the burden of stress and vital exhaustion are almost equally distributed between both treatment groups, a superiority of the SMT+BT training could not be derived. A previous study on chronic fatigue did show positive effects of exercise on fatigue symptoms and physical and psychological functioning in a small sample of women with chronic fatigue syndrome (Broadbent et al., 2018). However, in another study exercise reduced neck/shoulder pain but did not reduce work stress (Fanavoll et al., 2016). Both studies did not include multidisciplinary treatments, so the indication for people with stress and other psychological symptoms were missing. Further studies are clearly warranted to highlight the influence of multidisciplinary exercise-based treatments on low back pain and related symptoms of pain related cognitions, depression, anxiety, and stress.

Previous studies found that participants with low back pain decreased pain and disability at 12 and 24 months after interventions (motivation program; fear reduced and activity level increased program) (Friedrich et al., 1998; Von Korff et al., 2005). In addition, at 24 months of follow-up after the intervention (fear reduced and activity level increased program), the decrease of worry and fear avoidance still differ between groups (Von Korff et al., 2005). The different outcomes between the previous and the current study might be caused by the baseline value. At the baseline, participants in the previous studies showed a higher pain intensity (50–58 measured by numerical rating scale) than the current study (27–33 measured by CPI) (Friedrich et al., 1998; Von Korff et al., 2005). As well as pain disability, participants in the previous study showed a higher level of disability (Roland disability questionnaire [0–23]: 12.3–11.4 [high level]) than the current study (DISS [0-100]: 9.6–17.9 [low level]) (Korff et al., 1992; Roland and Fairbank, 2000; Von Korff et al., 2005). These studies included patients with chronic low back pain. In the methodological perspective, a higher pain level at baseline leads to a stronger pain reduction in the first months of intervention. The current study showed a small degree of pain reduction that may be valuable regarding chronic pain prevention, which is essential in people with intermitted low back pain as we had included in our study.

### Limitations

The limitations of the results presented in this study lie in the sample size of *n* = 84 subjects, which corresponds to 11.3% of the total sample of the initial exercise study. This leads to a limited representativeness of the examined sample and thus reduces the transferability of the results to the general population. 54.8% of the sample of one study site did participate in the long-term follow-up survey. In addition, a selection bias—and thus distorted answers, e.g. in a positive direction—is therefore probable and should be taken into account in the interpretation of the results. This further leads to different baseline pain levels in the groups, which may impair their comparability. Because of the small sample size, statistical power is reduced, especially when adjustments with additional variables are included into the analysis. Hence, possible effects could miss statistical significance and are therefore not interpreted further as was the case with pain related cognition scales or depression in this subsample. Also, the psychosocial risk and comorbidities were low in the observed sample. The surveys took place—on average—14 months after the completion of the initial study and it is likely that a majority of subjects did not continue properly with the home-based intervention by themselves. Moreover, no information regarding potential treatments during the 14-months follow-up time was available. In future studies, in order to statistically substantiate these indications, a longer follow-up period should be established directly, and a correspondingly large sample should be included.

## Conclusion

The results of this study indicate sustainable effects of a homebased motor control exercise intervention on back pain related disability. There are further hints of sustainable effects of the multidisciplinary intervention, as well as intervention effects on pain related cognitions and depression, however, these could not be confirmed statistically due to a lack of statistical power. In order to be able to deepen the analysis of possible emergence effects and to discriminate unimodal exercise interventions against multidisciplinary approaches, further studies are needed with larger samples and longer follow-up periods.

## Data Availability Statement

The raw data supporting the conclusions of this article will be made available by the authors, without undue reservation.

## Ethics Statement

The studies involving human participants were reviewed and approved by all surveys were designed and implemented in accordance with the principles of the Declaration of Helsinki and approved by the independent Ethics Committee of the University of Potsdam, Germany (Ethics Vote of the University of Potsdam, No. 19/2015). The patients/participants provided their written informed consent to participate in this study.

## Author Contributions

A-KP: writing original draft, methodology, and formal analysis. C-IL: editing and visualization. P-MW: conceptualization, data curation, investigation, and funding and project administration (PI). All authors reviewed the document and approved the final version for submission.

## Conflict of Interest

The authors declare that the research was conducted in the absence of any commercial or financial relationships that could be construed as a potential conflict of interest.

## Abbreviations

CPG: chronic pain grade questionnaire
CPI: chronic pain intensity
DISS: pain related disability
FABQ-D: fear avoidance beliefs questionnaire (German version)
HADS-D: hospital and anxiety depression scale
M: means
PSS: perceived stress scale
SD: standard deviation
SMT: unimodal intervention: sensorimotor training
SMT+BT: multidisciplinary intervention: sensorimotor training with elements of behavioral therapy
VE: Maastricht vital exhaustion questionnaire.
